# Economic burden of atherosclerotic cardiovascular disease: a matched case–control study in more than 450,000 Swedish individuals

**DOI:** 10.1186/s12872-023-03518-y

**Published:** 2023-09-29

**Authors:** Katarina Steen Carlsson, Kristoffer Nilsson, Michael Lyng Wolden, Mads Faurby

**Affiliations:** 1 The Swedish Institute for Health Economics (IHE), Lund, Sweden; 2 Department of Clinical Sciences, Malmö, Lund University, Lund, Sweden; 3 Novo Nordisk A/S, Søborg, Denmark

**Keywords:** Atherosclerotic cardiovascular disease, Burden of illness, Mortality, Direct costs, Indirect costs

## Abstract

**Aim:**

To examine direct and indirect costs, early retirement, cardiovascular events and mortality over 5 years in people with atherosclerotic cardiovascular disease (ASCVD) and matched controls in Sweden.

**Methods:**

Individuals aged ≥ 16 years living in Sweden on 01 January 2012 were identified in an existing database. Individuals with ASCVD were propensity score matched to controls without ASCVD by age, sex and educational status. We compared direct healthcare costs (inpatient, outpatient and drug costs), indirect costs (resulting from work absence) and the risk of stroke, myocardial infarction (MI) and early retirement.

**Results:**

After matching, there were 231,417 individuals in each cohort. Total mean per-person annual costs were over 2.5 times higher in the ASCVD group versus the controls (€6923 vs €2699). Indirect costs contributed to 60% and 67% of annual costs in the ASCVD and control groups, respectively. Inpatient costs accounted for ≥ 70% of direct healthcare costs. Cumulative total costs over the 5-year period were €32,011 in the ASCVD group and €12,931 in the controls. People with ASCVD were 3 times more likely to enter early retirement than controls (hazard ratio [HR] 3.02 [95% CI 2.76–3.31]) and approximately 2 times more likely to experience stroke (HR 1.83 [1.77–1.89]) or MI (HR 2.27 [2.20–2.34]).

**Conclusion:**

ASCVD is associated with both economic and clinical impacts. People with ASCVD incurred considerably higher costs than matched controls, with indirect costs resulting from work absence and inpatient admissions being major cost drivers, and were also more likely to experience additional ASCVD events.

**Supplementary Information:**

The online version contains supplementary material available at 10.1186/s12872-023-03518-y.

## Introduction

Cardiovascular disease (CVD) affected over 120 million people globally in 2016 [1]. The majority of cases are atherosclerotic CVD (ASCVD) [2, 3], which is characterized by the build-up of atherosclerotic plaques in the arteries [3, 4]. The major clinical manifestations of ASCVD are coronary/ischaemic heart disease, ischaemic stroke, and peripheral arterial disease (PAD) [3, 4], and ASCVD represents a leading cause of premature death worldwide [3–5].

Both CVD [6, 7] and ASCVD are specifically associated with significant morbidity and mortality [3, 8, 9], as well as a substantial economic burden [10–13]. Most of the available evidence on the economic burden of ASCVD focuses on healthcare resource utilization and the direct costs associated with both acute and long-term medical care [11–13], whereas few studies evaluate indirect costs at an individual level.

A small number of cost-of-illness studies have shown that ASCVD is associated with indirect costs due to lost productivity [10, 14–17]; however, these studies typically rely on imprecise aggregate data and are often based on small studies or expert opinion. Moreover, cost-of-illness studies typically present data on costs in one year for a prevalent population. From a policy perspective, the burden of ASCVD over time, comparing costs before and after onset, is another way of identifying the impact of ASCVD. Indeed, in people with diabetes, individual-level data show the high cost of work absence due to diabetes-related complications, such as ASCVD, especially in the first year after the event [18].

Population-based administrative registries hold large amounts of individual-level, real-world data on the occurrence of disease, healthcare resource use and work absence. In addition, using population-based registries often means that multiple data sources can be connected to allow exploration of the broader economic burden of common conditions. For example, linkage of healthcare resource use to subsequent mortality or work absence for people with defined diagnoses permits evaluation of outcomes over time and helps researchers to understand the wider societal impact of disease and interventions [9, 18–20]. Notably, these registries have been used to assess the impact of both ASCVD and more specific cardiovascular (CV) conditions on a wide range of outcomes, including hospital admission and mortality [9, 20, 21]. Recently, a research database was created in Sweden containing cross-linked individual-level health, social insurance and socioeconomic longitudinal data from individuals with and without diabetes. These data have previously been used to assess the impact of type 2 diabetes (T2D) complications on work absences and healthcare resource utilization [19, 20].

In the present study, we examined direct healthcare costs, indirect costs resulting from work absence, early retirement, stroke, myocardial infarction (MI) and mortality over 5 years, comparing individuals with ASCVD and matched controls without ASCVD. In a separate analysis, we examined the impact of the first occurrence of ASCVD on direct and indirect costs.

## Methods

### Study design and data sources

This observational, retrospective, closed-cohort study used an existing database at the Swedish Institute for Health Economics, originally set up to identify individuals with diabetes, plus control individuals without diabetes, between the ages of 16 and 70 years. Details of patient and control selection have been published previously [19, 20]. In brief, people aged between 16 and 70 years who met criteria for diabetes (see Supplementary Methods) at any point during 1997–2016 were selected for the database. For each person with diabetes, control participants from the general population with no record of diabetes were matched 5:1 using exact year of birth, sex and region of residence in the index year. This database was considered to provide good representation of the Swedish population with diabetes who were active in the labour market [20], and because control participants were matched for demographic factors, the control population can also be considered to reflect characteristics of the broader Swedish population in those demographic groups. Most importantly, the sample was large enough to allow identification of people for the control group who did not meet criteria for ASCVD. The database cross-links individual patient data (1997–2017) on prescription fills and healthcare resource utilization from the National Board of Health and Welfare (NBHW); demographic and socioeconomic data from Statistics Sweden; and data on work absences from Försäkringskassan (the Swedish Social Insurance Agency; Supplementary Table S1).

### Study population

Individuals aged 16 years or older living in Sweden on 01 January 2012 were identified in the full population of individuals with T2D (*n* = 491,116) plus controls (*n* = 2,423,626) in the existing database. ASCVD was defined as the presence of at least one inpatient admission or outpatient visit with a main or sub-diagnosis of cerebrovascular disease, ischemic heart disease, or PAD (see Supplementary Table S2 for ICD-10 codes) in the National Patient Register from 1997 to 31 December 2011. Baseline was 01 January 2012. Follow-up was until the end of the study period (2016), death or migration from Sweden, whichever was earliest. Individuals with ASCVD were propensity-score-matched 1:1 to individuals without ASCVD before 01 January 2012 in the database, with covariates for age, sex and educational status (a proxy for socioeconomic status) in 2012, using logistic regression with nearest neighbour matching. The matching variables were limited to demographic variables and socioeconomic status, meaning that potential differences in comorbidities before the baseline date (1997–2011) between individuals with similar demographic characteristics could be identified.

### Outcomes

Outcomes for individuals with ASCVD were compared with those for matched controls without any observed occurrence of ASCVD before 2012.

Total estimated costs in people with ASCVD were calculated as the sum of direct and indirect costs. Both mean annual costs per person and 5-year cumulative costs per person were calculated and are presented; mean cumulative costs were calculated among individuals alive in each separate year. Costs in Swedish krona were converted to euros using the average exchange rate for 2016.

Direct healthcare costs comprised inpatient admissions, hospital outpatient visits and prescribed drug costs. All hospital-based care for which any diagnostic code listed in Supplementary Table S3 was recorded as the main or secondary diagnosis was included. Inpatient admissions and hospital outpatient visits were costed using their respective diagnosis-related group (DRG) code and the national Swedish DRG tariff for year 2016 (the last study year). The total costs (both patient fee and publicly covered) of all filled prescriptions for selected drugs (anti-hypertensive, glucose-lowering and lipid-lowering agents) were obtained from the National Prescribed Drug Register using the Anatomic Therapeutic Chemical (ATC) classification system codes in Supplementary Table S4.

Indirect costs were calculated using data on days absent from work and average national earnings by age and sex in 2016. Days absent from work included registrations of full-time and part-time sickness benefits (short term absence, up to 1 year) and activity compensation (long term absence/early retirement). Indirect costs and early retirement were analysed for individuals aged under 66 years, i.e. labour-market active ages.

In addition to direct and indirect costs, the risk of four types of events were analysed and compared between the ASCVD and control groups: full-time early retirement, an inpatient admission due to stroke (ICD-10 codes I61, I63, I64 or I67.9) or MI (ICD-10 code I21), or death at any time during the study period. To ensure that the ASCVD-related diagnosis was the main cause of hospital admission, the analysis of events used a stricter definition to that used in the main analysis and required that stroke or MI was the main diagnosis for inpatient admission.

### Incident ASCVD analyses

Annual total, direct and indirect costs were assessed in a subset of individuals with their first observed occurrence of ASCVD in 2011 (the year before baseline). Mean annual per-person costs for the years 2007–2016 were compared with costs for controls to quantify the impact of incident ASCVD.

### Statistical methods

Mean and standard deviation (SD) are reported for continuous variables, and categorical variables are reported as actual numbers and proportions (%). Standardized differences for baseline characteristics were calculated after matching using the Stata package ‘stddiff’, and a standardized difference of greater than 0.1 was considered to indicate that groups were not balanced. Cost estimates are reported as mean and standard error (SE). Fine–Gray competing risk regression analyses (levels: 0 = censored; 1 = event-of-interest; 2 = death) were used to estimate the hazard ratios (HRs) and 95% confidence interval (CIs) for full-time early retirement, stroke, MI and death, with adjustments for non-CV conditions (diabetes, kidney disease and eye disease). Individuals who were already in early retirement before baseline were excluded from the estimation of the HR for full-time early retirement.

Potential violations of proportionality assumptions were tested using visual inspection of log–log plots and observed Kaplan–Meier plots against Cox predicted curves. Schoenfeld residuals were also calculated for each regression variable, with the null hypothesis of proportional hazards.

## Results

### Baseline characteristics

We identified 231,417 individuals with ASCVD in at least one related observation before the study period (01 January 2012). From a pool of 2,088,675 potential controls without ASCVD, 231,417 individuals were matched 1:1 based on age, sex and educational status. The numbers of individuals in the control group who subsequently met the criteria for ASCVD in each year following the start of the study are shown in Supplementary Table S5. These individuals were retained in the control groups.

Standardized differences of the matching variables indicate similarities between individuals with ASCVD and the controls (Table 1). In both groups, the mean age was 68 years (SD 7.7 years), 30% of individuals were women and 62% had upper secondary or university as their highest level of education. However, differences in the prevalence of comorbidities were observed between the groups. Individuals with ASCVD were more than twice as likely to have T2D than the controls (34% vs 14%), nearly 3 times more likely to have kidney disease (9.2% vs 3.2%) and 4 times more likely to have end-stage renal disease (1.2% vs 0.3%).

**Table 1:** Baseline characteristics for individuals with ASCVD and matched controls

**Characteristics**	**Individuals with ASCVD** **(*n* = 231,417)**	**Control individuals** **(*n* = 231,417)**	**Standardized differences**
**Propensity-matched characteristics**			
Age, mean (SD)	68.0 (7.7)	68.0 (7.7)	0.00
Women, n (%)	69,182 (29.9)	69,182 (29.9)	0.00
Education, n (%)			0.00
Compulsory	86,476 (37.4)	86,481 (37.4)	
Upper secondary	98,818 (42.7)	98,813 (42.7)	
University	43,937 (19.0)	43,946 (19.0)	
Missing	2186 (0.9)	2177 (0.9)	
**Early retirement, *n* (%)**			
At least part-time	91,485 (39.5)	50,225 (21.7)	0.39
Full-time	36,549 (15.8)	18,433 (8.0)	0.24
**ASCVD variables, *n* (%)**			
Ischaemic heart disease	150,119 (64.9)	0 (0.0)	1.92
Myocardial infarction	72,810 (31.5)	0 (0.0)	0.96
Cerebrovascular disease	58,259 (25.2)	0 (0.0)	0.82
Peripheral artery disease	21,389 (9.2)	0 (0.0)	0.45
Amputation	2776 (1.2)	0 (0.0)	0.16
**Comorbidities at baseline, *n* (%)**			
Type 2 diabetes	78,319 (33.8)	33,096 (14.3)	0.47
Atrial fibrillation	24,288 (10.5)	10,286 (4.4)	0.23
Heart failure	17,685 (7.6)	2540 (1.1)	0.32
Eye disease	78,478 (33.9)	59,553 (25.7)	0.18
Neuropathy	7992 (3.5)	5329 (2.3)	0.07
Kidney disease	21,228 (9.2)	7519 (3.2)	0.25
End-stage renal disease	2766 (1.2)	756 (0.3)	0.10

*ASCVD* atherosclerotic cardiovascular disease, *ICD-10-CM* International Classification of Diseases, Tenth Revision, Clinical Modification, *SD* standard deviation

### Total costs

**Fig. 1:**
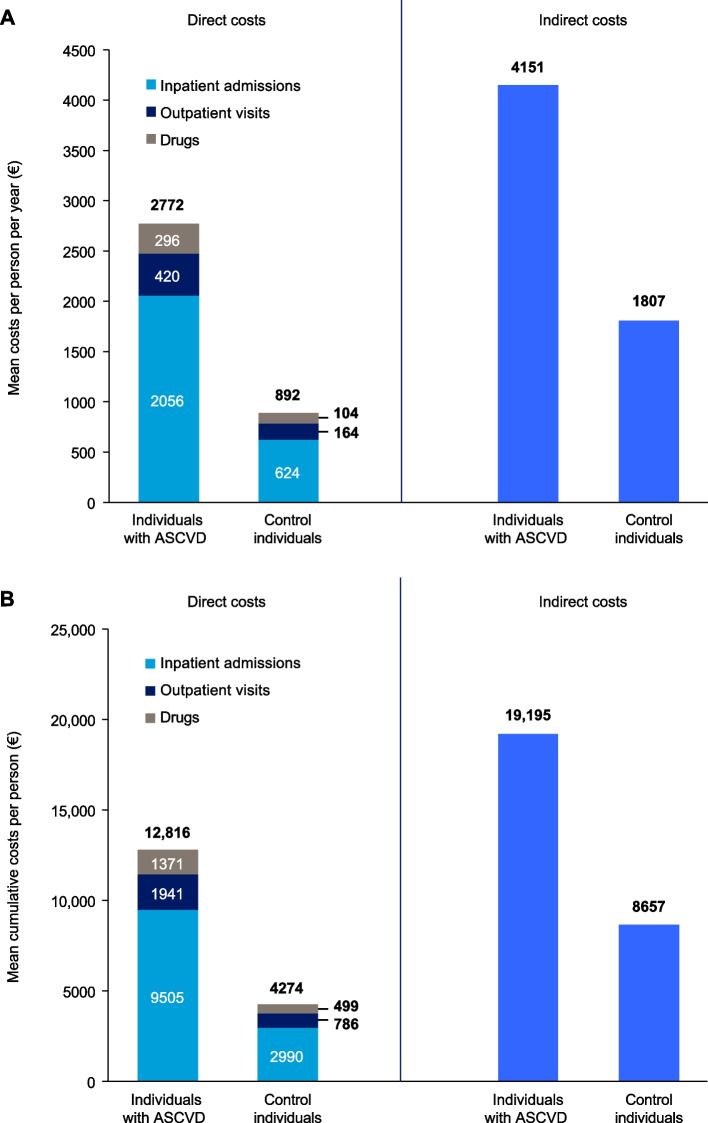
(**A**) Mean annual and (**B**) cumulative direct and indirect costs in individuals with ASCVD and controls over the 5-year study period. Mean annual and mean cumulative costs were higher in individuals with ASCVD compared with control individuals when looking at direct costs and indirect costs. In individuals with ASCVD, indirect costs were higher than direct costs (mean costs and mean cumulative costs); the same was also true for control individuals. Data are mean costs in the study period among all people, regardless of number of years alive. Individuals with ASCVD, *n* = 231,417; controls, *n* = 231,417. Indirect costs were calculated for the subsample of individuals aged < 66 years (individuals with ASCVD, *n* = 77,142; controls, *n* = 77,142). *ASCVD* atherosclerotic cardiovascular disease

Mean per-person annual costs were more than 2.5 times higher in the ASCVD group (€6923, standard error [SE] 15) than in the control group (€2699, SE 9), with a similar pattern for cumulative total per-person costs over 5 years (ASCVD €32,011 [SE 129]; control €12,931 [SE 81]). The greatest contributor to these totals was indirect costs, which contributed 60% of annual and 60% of cumulative costs in the ASCVD group (annual €4151 [SE 12]; cumulative €19,195 [SE 115]) and 67% of annual and cumulative costs in the control group (annual €1807 [SE 8]; cumulative €8657 [SE 76]; Fig. 1).

### Direct healthcare costs

Mean annual total direct costs were 3 times higher for individuals with ASCVD than for controls (€2772 [SE 8] vs €892 [SE 4]; Fig. 1A). In people with ASCVD, inpatient costs comprised 74% of total annual mean direct costs over the 5-year follow-up (€2056 [SE 7]). Outpatient costs contributed 15% (€420 [SE 3]) and drug costs contributed 11% (€296 [SE 0]) of total annual direct costs. In controls, inpatient costs contributed 70% (€624 [SE 4]), outpatient costs contributed 18% (€164 [SE 1]) and drug costs contributed 12% (€104 [SE 0]), respectively, of total annual mean direct costs.

**Fig. 2:**
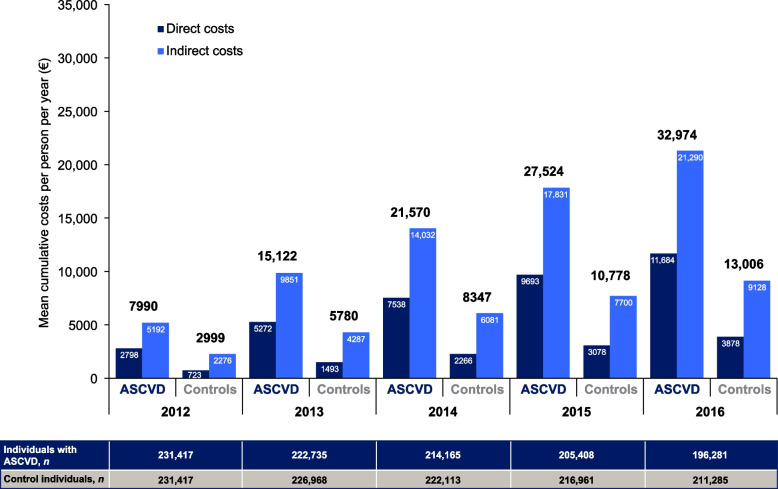
Mean cumulative costs per year in individuals with ASCVD and controls over the 5-year study period. In each year from 2012 to 2016, mean cumulative direct and indirect costs were higher in individuals with ASCVD compared with controls, with costs increasing each year. In each year, mean cumulative indirect costs were higher than mean cumulative direct costs. *ASCVD* atherosclerotic cardiovascular disease

Over the 5-year follow-up period, cumulative direct costs for people with ASCVD remained consistently higher than those for controls (Fig. 2 and Supplementary Table S6). In the index year, costs were nearly 4 times higher for individuals with ASCVD than for controls (€2798 vs €723), and in the last year of the study period, they remained more than 3 times higher (€11,684 vs €3878). Cumulative 5-year direct costs were €12,816 (SE 51) per person for individuals with ASCVD and €4274 (SE 25) for controls (Fig. 1B).

### Indirect costs from work absence

Work absences and resultant indirect costs were calculated for working age people younger than 66 years in each year. There were 77,142 individuals in each group in 2012. Age, death and emigration reduced the sample to 39,867 individuals with ASCVD and 41,355 controls in 2016. Mean annual total indirect costs per person were 2.3 times higher in those with ASCVD than in controls (€4151 [SE 12] vs €1807 [SE 8]; Fig. 1A), and 5-year cumulative costs were almost double (€19,195 [SE 115] vs €8657 [SE 76]; Fig. 1B). The disparity between groups remained relatively stable over the study period: indirect costs per person were about 1.8–2.3 times higher for individuals with ASCVD than for controls in both 2012 (€5192 [SE 29] vs €2276 [SE 19]) and 2016 (€3,182 [SE 25] vs €1807 [SE 8]), (Fig. 2 and Supplementary Table S6).

The average number of days absent from work per year was more than double in individuals with ASCVD (116 days) compared with controls (52 days). For those with ASCVD, on average, 8% were absent for 180–269 days per year and 17% were fully absent each year. For controls, 4% were absent for 180–269 days and 7% for more than 360 days per year. Among individuals with ASCVD, 52% had no work absence in the year 2012, compared with 75% of controls.

### Costs following first observed occurrence of ASCVD

**Fig. 3:**
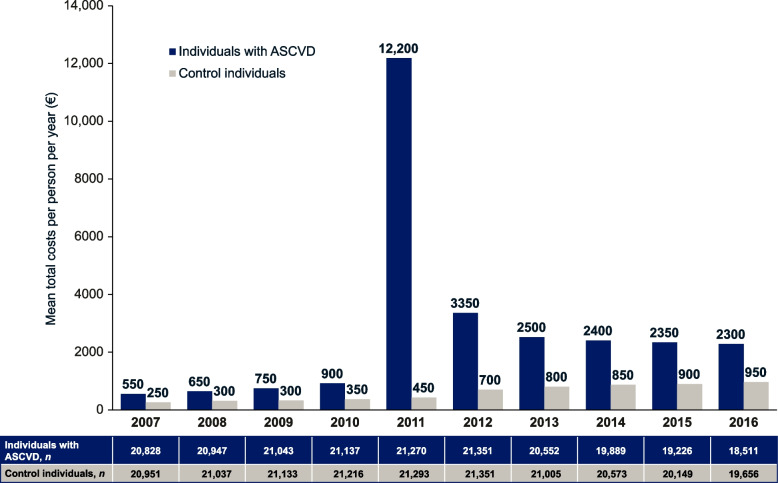
Mean direct costs over the 5 years before and after the first occurrence of ASCVD (2011) in individuals with ASCVD compared with controls. In individuals with ASCVD, mean direct costs increased gradually from 2007 to 2010, and then displayed a spike in costs in 2011 when ASCVD first occurred. In the year after the first event (2012), mean direct costs were lower than they had been in the previous year (2011) and stabilized at a higher level (2012–2016) than they had been before the ASCVD event (2007–2010). In the controls, mean indirect costs were lower than in individuals with ASCVD, and they gradually increased over time, from 2007 to 2016. Total costs are rounded to the nearest €50. *ASCVD* atherosclerotic cardiovascular disease

For the subgroup of individuals with a first observed occurrence of ASCVD in 2011, the year before the study period (*n* = 21,351 in 2012), annual costs were examined from 2007 to 2016 and compared with controls. Total costs decreased gradually decreased from 2007 to 2010 in individuals with observed ASCVD but increased sharply in 2011, from €6263 in 2010 to €18,538 in 2011. This increase in the ASCVD group was mostly driven by direct costs, which increased from €920 in 2010 to €12,188 in 2011 (Fig. 3), and in particular, inpatient costs, which increased from €533 in 2010 to €11,229 in 2011 (Supplementary Table S7). For the remainder of follow-up, direct costs decreased but remained higher than they had been in the year before ASCVD was first recorded.

Indirect costs decreased year by year over the follow-up period in both individuals with ASCVD and the controls, except in the year immediately following the occurrence of ASCVD, during which there was an increase from the preceding year (2010 €5343; 2011 €6350). The trend for a decrease in indirect costs over time is likely to be the result of population aging, whereby the proportion of individuals reaching retirement age counteracts the impact of work absence on costs.

### CV events and mortality

**Fig. 4:**
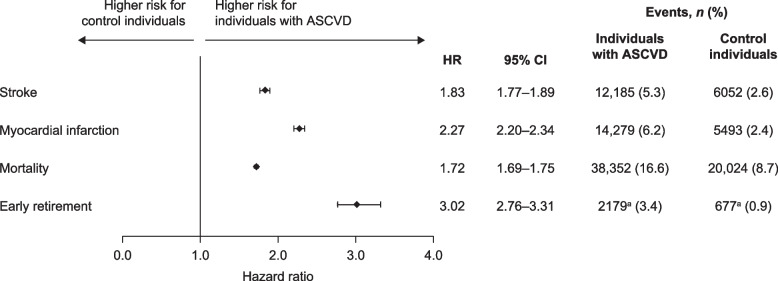
Hazard ratios for cardiovascular events, mortality and early retirement. The hazard ratios for stroke, myocardial infarction, mortality and early retirement indicated that the risk of these events were higher in individuals with ASCVD compared with controls. Individuals with ASCVD, *n* = 231,417; control individuals, *n* = 231,417. ^a^Includes only individuals younger than 66 years old and not already in early retirement in 2012 (individuals with ASCVD, *n* = 63,480; control individuals, *n* = 71,522). *ASCVD* atherosclerotic cardiovascular disease, *CI* confidence interval, *ICD-10* International Classification of Diseases, Tenth Revision

People with ASCVD were more likely to experience stroke (12,185 vs 6052 events; HR 1.83 [95% CI 1.77–1.89]) or MI (14,279 vs 5493 events; HR 2.27 [95% CI 2.20–2.34]) than their controls (Fig. 4). During the study period, 17% (*n* = 38,352) of individuals in the ASCVD cohort died, compared with 8.7% (*n* = 20,024) of controls, and all-cause mortality was 1.7 times higher (HR 1.7 [95% CI 1.7–1.8]) in those with ASCVD than in controls (Fig. 4).

Log–log plots and assessment of Kaplan–Meier versus Cox curves indicated that proportional hazards assumptions had not been violated. Schoenfeld residuals testing indicated some violations of proportional hazards assumptions; however, this is to be expected in a large sample. Altogether, the proportionality was considered to be reasonable within the time horizon of the study.

### Early retirement

At baseline, 16% of individuals with ASCVD were in full-time early retirement compared with 8.0% of controls (Table 1). Individuals with ASCVD were more than 3 times more likely to enter full-time early retirement during the study period than controls (HR 3.02 [95% CI 2.76–3.31]; Fig. 4).

## Discussion

This study demonstrates that people with ASCVD in Sweden incur considerable costs arising from direct costs of hospital-based care and indirect costs resulting from work absence. We followed a large population with ASCVD over a 5-year period, and compared them with matched controls without ASCVD from the general population. An analysis of incident ASCVD also highlighted a sizeable and sustained increase in direct healthcare costs immediately after the occurrence of ASCVD.

On average, total costs were approximately 2.5 times higher in the ASCVD group than in the control group. Inpatient costs were the main contributors to the mean annual per-person direct costs in individuals with ASCVD, suggesting that hospitalization has a bigger impact on the economic burden of disease than the costs associated with longer-term care, such as outpatient visits and cost of prescription drugs. However, indirect costs resulting from work absence were the largest overall cost driver, contributing 60% of mean annual per-person total costs in the group with ASCVD and 67% of total costs in controls. This contrasts with recent studies on the economic impact of more broadly defined CVD, which have reported higher direct costs than indirect costs in the USA [1], and an approximately equal contribution of direct and indirect costs to total costs in the European Union [6]. These disparities may reflect differences between populations, data sources, countries’ retirement policies, or study methodology, including disease definitions. For example, some studies may have a broader definition of CVD that includes hyperlipidaemia and hypertension and so their population may have less severe disease than the individuals included in our study. The lower indirect costs observed in other studies may also be because they are reliant on data from surveys or clinical trial follow-up and lack long-term data on work absence. Some studies also report work absence as a binary variable, not considering the difference between sick leave, which is temporary, and early retirement, which is permanent.

Individuals with ASCVD had nearly 2.5 times higher indirect costs than the controls, missed twice as many workdays and were more likely to retire early. These findings may be due to some of the specific clinical manifestations of ASCVD. For example, a study of individuals who experienced ischaemic stroke found that fewer than 50% returned to work within 6 months [22], while a systematic literature review assessing the impact of stroke found that more than 30% of affected individuals had not returned to work up to 4 years afterwards [23]. PAD has also been associated with reduced mobility [3], which can, in turn, increase absenteeism. Although indirect costs were the biggest contributor to the overall economic burden, ASCVD occurrence had a much larger immediate impact on direct healthcare costs, which were over 13 times higher in the year ASCVD was first observed than in the year before, and remained elevated above baseline levels thereafter. This highlights the need for acute medical care and inpatient treatment following ASCVD occurrence. ASCVD was also associated with an increased risk of CV events over 5 years, compared with the control group, indicating another potential driver of direct costs. Finally, the baseline prevalence of comorbidities was higher in the ASCVD group compared with the controls, indicating that individuals with ASCVD were more likely than controls to have other cardiometabolic conditions, such as T2D, and kidney disease, which are also associated with high inpatient costs [13, 19, 24–26].

This study made use of linked mortality, early retirement, work absence and healthcare data from several national health and social insurance registers with well-established procedures for making individual-level data available for research. This allowed estimation of indirect costs for people with ASCVD, which, to the best of our knowledge, have not been widely reported in the published literature, owing to limited data availability [11–13]. Furthermore, the large sample size available permitted propensity score matching of control individuals, to reduce bias arising from disparities in demographic characteristics. Individuals were matched with similar controls for sex, age and education, which is often used as a marker for socioeconomic status, but not for comorbidities. This resulted in a population with ASCVD with a greater overall comorbidity burden than the control population at baseline, meaning that the costs observed in this ASCVD group cannot be considered solely the result of ASCVD, but may also be attributable to concomitant conditions. By capturing the association between ASCVD and other conditions, this allows assessment of the full economic and clinical burden associated with ASCVD. In addition, not matching based on comorbidities means that the controls reflect the general population of the same age, sex and level of education better than if this matching on comorbidities had been performed. This is because ASCVD is relatively common and therefore identifying controls with similar demographic characteristics and comorbidities would have been challenging. Our data provide a comprehensive assessment of the overall health, mortality, participation in the workforce and economic costs, borne by healthcare systems and society, for people with ASCVD. Assessment of the precise impact of ASCVD on costs and risks of CV events, excluding the influence of commonly co-occurring conditions, was outside the scope of our study, but would be a valuable approach in future analyses.

This study was designed with the intention of capturing the burden on patients with established ASCVD who have experienced at least one ASCVD event (i.e. the secondary ASCVD prevention population) by including individuals who had at least one registered occurrence of ASCVD at a hospital. The primary prevention population (patients who have not experienced an ASCVD event but have early, sub-clinical stages of ASCVD or risk factors for ASCVD) may also benefit from preventative interventions and should be evaluated in future studies. A limitation of this study concerns the fact that the existing database only included individuals with diabetes onset before the age of 70 years and their controls, and that although follow-up spanned up to 20 years, the dataset is likely to be under-representative of the very elderly section of the general population. Data on primary care were not available for this analysis and, although hospital costs are expected to be a comparatively far larger contributor to the overall economic burden of CVD, this means that the total costs in this study are likely to be underestimated. The contribution of primary care to healthcare costs is also likely to vary between different manifestations of ASCVD; further investigation of this is warranted.

This study demonstrates that ASCVD incurs considerable economic costs, both immediately after the first occurrence and over the subsequent 5 years. Work absence and inpatient admissions are major drivers of costs, with drug and outpatient visits only contributing to a small part of the total healthcare costs associated with ASCVD. ASCVD is a common condition, and its effects on individuals, society and healthcare systems should be a key consideration in treatment decision-making and healthcare policy. ASCVD is strongly linked to modifiable risk factors [3, 4, 8], and initiatives focused on the management of these will help to alleviate or at least postpone the impacts of disease development and progression.

### Supplementary Information

**Additional file 1:** **Supplementary Methods.** **Supplementary Table S1.** Data sources and variables included. **Supplementary Table S2.** ICD-10 codes for the broad definition of ASCVD. **Supplementary Table S3.** ICD-10 codes and hospital procedure codes (KVÅ) selected from the National Patient Register. **Supplementary Table S4**. ATC codes selected from the National Prescribed Drug Register. **Supplementary Table S5.** Numbers of people in the control group who met the criteria for ASCVD in each year of the study. **Supplementary Table S6.** Cumulative total, direct and indirect cost data for individuals with ASCVD and control individuals. **Supplementary Table S7.** Mean annual direct costs (€) before and after the first observed occurrence of ASCVD in 2011.  

## Data Availability

Subject-level data from national registers hosted by Swedish national authorities are available for research after formal evaluation of the research protocol by the Swedish Ethical Review Authority and by the respective national authorities providing data. Permission to conduct research is granted on a case-by-case basis to a limited number of named people. The secondary individual-level data of the study cannot be shared by the authors for this reason. Related aggregated summary data from the subject-level data of the current study are available from the corresponding author on reasonable request.
