# Supplementary material for: Economic burden of atherosclerotic cardiovascular disease: a matched case–control study in more than 450,000 Swedish individuals
Source: BMC Cardiovasc Disord. 2023 Sep 29;23:483. doi: 10.1186/s12872-023-03518-y (PMC10540350; doi:10.1186/s12872-023-03518-y)
Supplement: Supplementary file 1 — Additional file 1: Supplementary Methods. Supplementary Table S1. Data sources and variables included. Supplementary Table S2. ICD-10 codes for the broad definition of ASCVD. Supplementary Table S3. ICD-10 codes and hospital procedure codes (KVÅ) selected from the National Patient Register. Supplementary Table S4. ATC codes selected from the National Prescribed Drug Register. Supplementary Table S5. Numbers of people in the control group who met the criteria for ASCVD in each year of the study. Supplementary Table S6. Cumulative total, direct and indirect cost data for individuals with ASCVD and control individuals. Supplementary Table S7. Mean annual direct costs (€) before and after the first observed occurrence of ASCVD in 2011. [file 12872_2023_3518_MOESM1_ESM.docx]

Economic burden of atherosclerotic cardiovascular disease: a matched case–control study in more than 450,000 Swedish individuals

**Authors**

Katarina Steen Carlsson^1,2*^ • Kristoffer Nilsson^1^ • Michael Lyng Wolden^3^ • Mads Faurby^3^

**Author affiliations**

^1^The Swedish Institute for Health Economics (IHE), Lund, Sweden

^2^Department of Clinical Sciences, Malmö, Lund University, Lund, Sweden

^3^Novo Nordisk A/S, Søborg, Denmark

***Corresponding author**

Email address: [katarina.steen_carlsson@ihe.se](mailto:katarina.steen_carlsson@ihe.se)

ORCID (if available): 0000-0002-2325-5634

# Supplementary Information

## Supplementary Methods

In the database at the Swedish Institute for Health Economics, individuals with diabetes were identified by the presence of at least one healthcare visit or inpatient stay with diabetes as the main or secondary diagnosis (ICD, Tenth revision [ICD-10] codes) between 1997 and 2016 in the National Patient Register, and/or at least two glucose lowering medication prescriptions (Anatomical Therapeutic Chemical [ATC] codes A10, ATC-A10) not more than 6 months apart between 1 July 2005 and 31 December 2017 in the Swedish Prescription Drug Register. Individuals with a code for receipt of insulin (ATC code A10A) in the National Prescribed Drug Register were considered to have either type 1 diabetes or type 2 diabetes, but those with at least one filled prescription for a drug with ATC code A10B (other glucose lowering medications) between July 2005 and December 2017 were considered to have type 2 diabetes.

## Supplementary Table S1 Data sources and variables included

| **Authority** | **Database** | **Variables** |
| --- | --- | --- |
| National Board of Health and Welfare | National Patient Register | Inpatient admissions to hospital care and outpatient visits to hospital-based specialist care  Variables include date of visit, date of admission, date of discharge, main and secondary diagnosis, intervention procedures and classification by diagnosis-related group |
|  | National Prescribed Drug Register | Filled prescriptions of medications in outpatient care  Variables include medication (product ATC code), date of filled prescription, volume and cost |
|  | Causes of Death Register | Year of death |
| Försäkringskassan [Swedish Social Insurance Agency] | Micro Data for the Analysis of Social Insurance | Days absent from work covering schemes of sickness and rehabilitation benefits, and sickness and activity compensation  Variables include dates of start and end, full-time and percentage degree of part-time absence |
| Statistics Sweden | Total Population Register | Year of birth, year of death, date of immigration and date of emigration |
|  | Longitudinal integrated database for health insurance and labour market Studies | Demographic and socioeconomic characteristics including level of education, marital status and family status |

*ATC* Anatomic Therapeutic Chemical classification system.

## Supplementary Table S2 ICD-10 codes for the broad definition of ASCVD

|  | **ICD-10 codes** | **Procedure codes** | **Reference publications** |
| --- | --- | --- | --- |
| Cerebrovascular disease | I61, I63, I64, I67 | – | [1-5] |
| Ischemic heart disease | I20–I25 | Codes related to percutaneous coronary intervention (PCI): FNG0, FNG00, FNG02, FNG05, FNG06, FNG10, FNG30, FNG96  Codes related to coronary artery bypass graft (CABG): FNA0, FNA00, FNA10, FNA20, FNA96, FNB00, FNB20, FNB96, FNC10, FNC20, FNC30, FNC40, FNC50, FNC60, FNC96, FND10, FND20, FND96, FNE00, FNE10, FNE20, FNE96, FNF00, FNF10, FNF20, FNF30, FNF96 | [1-4, 6] |
| Peripheral artery disease | I70.2, I73.1, I73.9, 179.2 | Codes related to amputation: NEQ19, NEQ99, NFQ09, NFQ19, NFQ99, NGQ09, NGQ19, NGQ99, NHQ09, NHQ11, NHQ12, NHQ13, NHQ14, NHQ16, NHQ17, NHQ99 | [1, 3, 4] |

*ASCVD* atherosclerotic cardiovascular disease, *CABG* coronary artery bypass graft, *ICD-10* International Classification of Diseases, Tenth Revision. *PCI* percutaneous coronary intervention.

## Supplementary Table S3 ICD-10 codes and hospital procedure codes (KVÅ) selected from the National Patient Register

| **Conditions and events** | **ICD 10 codes or procedural codes** |
| --- | --- |
| Type 2 or unspecified diabetes | E11, E14 |
| Angina pectoris | I20 |
| Acute myocardial infarction | I21 |
| Ischemic heart disease | I22–I25 |
| Atrial fibrillation | I48 |
| Heart failure | I50 |
| Stroke | I61, I63, I64, I67.9 |
| Periphery vascular disease (PVD) | I70.2, I73.1, I73.9, I79.2, E11.5, E14.5 |
| Lower extremity amputation | KVÅ: NEQ19, NEQ99, NFQ09, NFQ19, NFQ99, NGQ09, NGQ19, NGQ99, NHQ09, NHQ11, NHQ12, NHQ13, NHQ14, NHQ16, NHQ17, NHQ99 |
| Other sudden death, cause unknown | R96.0, R96.1 |
| Diabetic foot including ulcer | E11.6, E14.4, L00, L03, L08, L97 |
| Eye disease and diabetic retinopathy | H0–H4, H50–H53, H55, H57–H59, E103, E113, E143  KVÅ: DT006 |
| Severe vision loss/blindness on one or two eyes | H54 |
| Symptomatic neuropathy | E11.4D, G62.9, G56, G58.9, G99, G63.2, G73.3, M14.6 |
| Kidney disease (initial stages) | E11.2, E14.2, E11.7, E11.8, E14.7, E14.8, R809, N00–N08, N10–N19, N28.9 |
| End-stage renal disease (ESRD) | Dialysis: Z49, Z94, Z99.2  KVÅ Dialysis: DR015, DR016, DR017  KVÅ Transplantation: KAS10, KAS20, VF420 |
| Hypo- and hyperglycaemia including coma | E11.0, E11.6, E14.0, E14.6, R40.2, E16.0, E16.1, E16.2, R73.9 |
| Hyperglycaemia | R73.9, E11.0, E11.1, E14.0, E14.1 |
| Ketoacidosis with/without coma | E11.0A, E11.1A, E14.0, E14.1, R40.2 |
| Hypoglycaemia without/with coma | E11.0, E11.0C, E11.6, E11.6A, E14.0, E14.0C, E14.6A, R40.2, E16.0, E16.1, E16.2 |
| Coma (unspecified) | E11.0, E14.0, R40.2 |
| Osteoarthritis | M15–M19 |

Hospital procedure codes are defined according to the system of KVÅ, which includes surgical and non-surgical interventions, including diagnostic procedures.

*ESRD* end-stage renal disease, *ICD-10* International Classification of Diseases, Tenth Revision, *KVÅ* Klassifikation av vårdåtgärder [Classification of healthcare interventions], *PVD* periphery vascular disease.

## Supplementary Table S4 ATC codes selected from the National Prescribed Drug Register

| **Condition** | **ATC code (7 digit level) for medication** |
| --- | --- |
| Diabetes | A10 |
| Hypertension | C03, C07, C08, C09 |
| Dyslipidaemia | C10 |
| Retinopathy | S01LA04 |
| Neuropathy | N06AB, N06AA09, N03AX12, N03AX16, N06AX21 |

*ATC* Anatomic Therapeutic Chemical classification system.

## Supplementary Table S5 Numbers of people in the control group who met the criteria for ASCVD in each year of the study

| **Year** | **Number of controls who met criteria for ASCVD in calendar year** | **Number of controls in calendar year** | **Proportion of control group who met criteria for ASCVD in calendar year (%)** | **Prevalent cumulative number of people with ASCVD in the control group** |
| --- | --- | --- | --- | --- |
| 2012 | 4499 | 231,417 | 1.9 | 4499 |
| 2013 | 4311 | 226,968 | 1.9 | 8390 |
| 2014 | 4071 | 222,113 | 1.8 | 11,829 |
| 2015 | 4173 | 216,961 | 1.9 | 15,156 |
| 2016 | 3942 | 211,285 | 1.9 | 18,009 |

*ASCVD* atherosclerotic cardiovascular disease.

## Supplementary Table S6 Cumulative total, direct and indirect cost data for individuals with ASCVD and control individuals

|  | **Total costs** | | **Direct costs** | | **Indirect costs** | |
| --- | --- | --- | --- | --- | --- | --- |
| **Year** | **Individuals with ASCVD** | **Control individuals** | **Individuals with ASCVD** | **Control individuals** | **Individuals with ASCVD** | **Control individuals** |
| **2012** | 7990 (34) | 2999 (21) | 2798 (17) | 723 (8) | 5192 (29) | 2276 (19) |
| **2013** | 15,122 (64) | 5780 (39) | 5272 (27) | 1493 (13) | 9851 (56) | 4287 (37) |
| **2014** | 21,570 (93) | 8347 (56) | 7538 (37) | 2266 (17) | 14,032 (82) | 6081 (53) |
| **2015** | 27,524 (120) | 10,778 (72) | 9693 (45) | 3078 (21) | 17,831 (107) | 7700 (68) |
| **2016** | 32,974 (146) | 13,006 (87) | 11,684 (52) | 3878 (25) | 21,290 (132) | 9128 (82) |
| **5-year total (*n* = 231,417)** | 32,011 (129) | 12,931 (81) | 12,816 (51) | 4274 (25) | 19,195 (115) | 8657 (76) |

All data are mean (SE).

*ASCVD* atherosclerotic cardiovascular disease, *SE* standard error.

## Supplementary Table S7 Mean annual direct costs (€) before and after the first observed occurrence of ASCVD in 2011

|  | **Inpatient admissions,** € | | **Outpatient visits,** € | | **Prescribed drug costs,** € | |
| --- | --- | --- | --- | --- | --- | --- |
| **Year** | **Individuals with ASCVD** | **Control individuals** | **Individuals with ASCVD** | **Control individuals** | **Individuals with ASCVD** | **Control individuals** |
| 2007 | 262 (15) | 114 (9) | 114 (9) | 43 (4) | 181 (2) | 101 (2) |
| 2008 | 325 (15) | 149 (13) | 126 (10) | 57 (4) | 192 (3) | 108 (2) |
| 2009 | 393 (18) | 147 (11) | 157 (13) | 66 (6) | 199 (3) | 111 (2) |
| 2010 | 533 (22) | 196 (12) | 190 (15) | 66 (5) | 197 (3) | 109 (2) |
| 2011 | 11,229 (91) | 234 (13) | 680 (19) | 89 (7) | 280 (3) | 109 (2) |
| 2012 | 2544 (52) | 494 (23) | 542 (21) | 103 (7) | 275 (3) | 101 (2) |
| 2013 | 1836 (49) | 558 (29) | 442 (21) | 153 (8) | 245 (3) | 98 (2) |
| 2014 | 1739 (47) | 603 (26) | 412 (19) | 167 (8) | 253 (3) | 101 (2) |
| 2015 | 1695 (44) | 629 (27) | 383 (18) | 164 (7) | 262 (3) | 106 (2) |
| 2016 | 1626 (43) | 641 (25) | 387 (19) | 182 (8) | 268 (3) | 111 (2) |

All data are mean (SE).

*ASCVD* atherosclerotic cardiovascular disease, *SE* standard error.

## References

1. Adamsson Eryd S, Svensson AM, Franzén S, et al. Risk of future microvascular and macrovascular disease in people with Type 1 diabetes of very long duration: a national study with 10‐year follow‐up. Diabet Med. 2017;34(3):411–8. <https://doi.org/10.1111/dme.13266>.

2. Kiadaliri AA, Gerdtham U-G, Nilsson P, et al. Towards renewed health economic simulation of type 2 diabetes: risk equations for first and second cardiovascular events from Swedish register data. PLoS ONE. 2013;8(5):e62650. <https://doi.org/10.1371/journal.pone.0062650>.

3. Lung TW, Petrie D, Herman WH, et al. Severe hypoglycemia and mortality after cardiovascular events for type 1 diabetic patients in Sweden. Diabetes Care. 2014. <https://doi.org/10.2337/dc14-0405>.

4. Persson S, Johansen P, Andersson E, et al. Days absent from work due to complications associated with type 2 diabetes: Evidence from 20 years of linked national registry data in Sweden. Diabetes Obes Metab. 2020. 10.1111/dom.14070.

5. Tancredi M, Rosengren A, Svensson A-M, et al. Excess mortality among persons with type 2 diabetes. New Engl J Med. 2015;373(18):1720–32. <https://doi.org/10.1056/NEJMoa1504347>.

6. Steineck I, Cederholm J, Eliasson B, et al. Insulin pump therapy, multiple daily injections, and cardiovascular mortality in 18,168 people with type 1 diabetes: observational study. BMJ. 2015;350:h3234. <https://doi.org/10.1136/bmj.h3234>
